# Influence of hyaluronic acid on intra-articular friction – a biomechanical study in whole animal joints

**DOI:** 10.1186/s12891-022-05867-9

**Published:** 2022-10-20

**Authors:** Moritz Mederake, Dominik Trappe, Christopher Jacob, Ulf Krister Hofmann, Daniel Schüll, Philipp Dalheimer, Lisanne Exner, Christian Walter

**Affiliations:** 1 Department of Trauma and Reconstructive Surgery, BG Klinik, University of Tübingen, Schnarrenbergstraße 95, 72076 Tübingen, Germany; 2 University Hospital Tübingen, Hoppe Seyler –Str. 3, 72076 Tübingen, Germany; 3 Department of Orthopedic Trauma and Reconstructive Surgery, University of Aachen Medical Center, Pauwelsstraße 30, 52074 Aachen, Germany

**Keywords:** Hyaluronic acid, Friction, Biomechanics, Osteoarthritis, Dissipated energy, Biotribology

## Abstract

**Background:**

Cartilage is a mechanically highly stressed tissue in the human body and an important part of synovial joints. The joint cartilage is lubricated by synovial fluid with hyaluronic acid (HA) as main component. However, in joints with osteoarthritis HA has a lower concentration and molecular weight compared to healthy joints. In recent years, the intra-articular injection of therapeutic HA lubricant, has become a popular therapy. The effect of HA application on the friction of a complete joint with physiological movement needs to be further determined.

**Methods:**

The aim of the present study was to evaluate the lubrication effect of the joint by three lubricants (NaCl, fetal calf serum (FCS) and HA) and their effect on the friction in nine complete ovine carpo-metacarpal joints. The joints were mounted on a material testing machine and a physiological movement with 10° rotation was simulated with ascending axial load (100 – 400 N). Specimens were tested native, with cartilage damage caused by drying out and relubricated. Dissipated energy (DE) as a measure of friction was recorded and compared.

**Results:**

Investigating the effect of axial load, we found significant differences in DE between all axial load steps (*p* < .001), however, only for the defect cartilage. Furthermore, we could document an increase in DE from *native* (Mean: 15.0 mJ/cycle, SD: 8.98) to *cartilage damage* (M: 74.4 mJ/cycle, SD: 79.02) and a decrease after relubrication to 23.6 mJ/cycle (SD: 18.47). Finally, we compared the DE values for *NaCl*, *FCS* and *HA*. The highest values were detected for *NaCl* (M_Norm_ = 16.4 mJ/cycle, SD: 19.14). *HA* achieved the lowest value (M_Norm_ = 4.3 mJ/cycle, SD: 4.31), although the gap to *FCS* (M_Norm_ = 5.1 mJ/cycle, SD: 7.07) was small.

**Conclusions:**

We were able to elucidate three effects in joints with cartilage damage. First, the friction in damaged joints increases significantly compared to native joints. Second, especially in damaged joints, the friction increases significantly more with increased axial load compared to native or relubricated joints. Third, lubricants can achieve an enormous decrease in friction. Comparing different lubricants, our results indicate the highest decrease in friction for HA.

## Background

Cartilage is a mechanically highly stressed tissue in the human body, covering the bone ends and being an important part of synovial joints. It has a high water entrapping capacity and therefore functions as a shock absorber, due to the structure of the tissue and intermolecular interactions among polymeric components [1]. The lubrication of the cartilage and the synovial joint is supported by synovial fluid (SF), which is crucial for reducing the friction in the joint [2]. The main component of SF in a healthy joint is hyaluronic acid (HA) [3]. Further postulated key components for reduction of friction are proteoglycan 4 (PRG4) and surface-active phospholipids (SAPL), which are also mainly produced in synoviocytes [4, 5]. However, in joints with osteoarthritis HA has a lower concentration and molecular weight (MW) compared to healthy joints [6]. Effects of HA like scavenger functions, regulation of cellular activities, space filling and especially lubrication can therefore be impaired [2]. In recent years, the intra-articular injection of a therapeutic HA lubricant, involving the replenishment of the SF, has become a popular therapy for mild OA [7]. Clinical studies found a pain reduction in mild OA of the knee joint up to 24 weeks after intra-articular injection of HA [8]. Based on frequent use, various clinical studies examined the effect of HA injections in the knee joint on symptoms such as pain reduction or joint mobility [9–14]. Molecular effects of the replenishment of HA are the increase of proteoglycan synthesis, suppression of the production of inflammatory mediators and the influence on immune cells [15].

The original rationale for the therapeutic application of HA in OA was to increase the viscosity of SF and the reduction of friction [15]. Therefore, a biomechanical investigation of the HA application is obvious. Existing studies of the cartilage lubrication, friction and the effects of HA are limited to pin-on-disc [16–18] and pendulum [19] measurements or to non-physiological joint movements [20]. Effects on friction are promising, however, the effect of HA application on the friction of a complete joint with physiological movement has not been investigated in previous studies.

The determination of the dissipated energy (DE) as a parameter for the friction in whole animal joints has been established in recent years [21]. Various changes in the cartilage surface such as local cartilage defect [22, 23], repositioning inaccuracies in tibial head fractures [24] or osteochondral transplantation [25, 26] and their effect on DE are examined and characterized.

The aim of the present study was to evaluate the lubrication of the joint by various substances and their effect on the friction using the DE in complete joints. Similar to previous studies, we used ovine carpo-metacarpal (CMC) joints, due to their standardized physiologic rotational movement. First, we asked whether the dependence of the DE on the axial load, which has been documented in previous studies, can be reproduced in the lubrication environment. Second, we investigated whether the DE can be increased through artificial joint damage and then reduced again through lubrication. Finally, we determined whether HA leads to an improved reduction in friction compared to other lubricants.

## Methods

### Specimens

The study was conducted according to the Directive 2010/63/EU and the Basel Declaration.

**Fig. 1:**
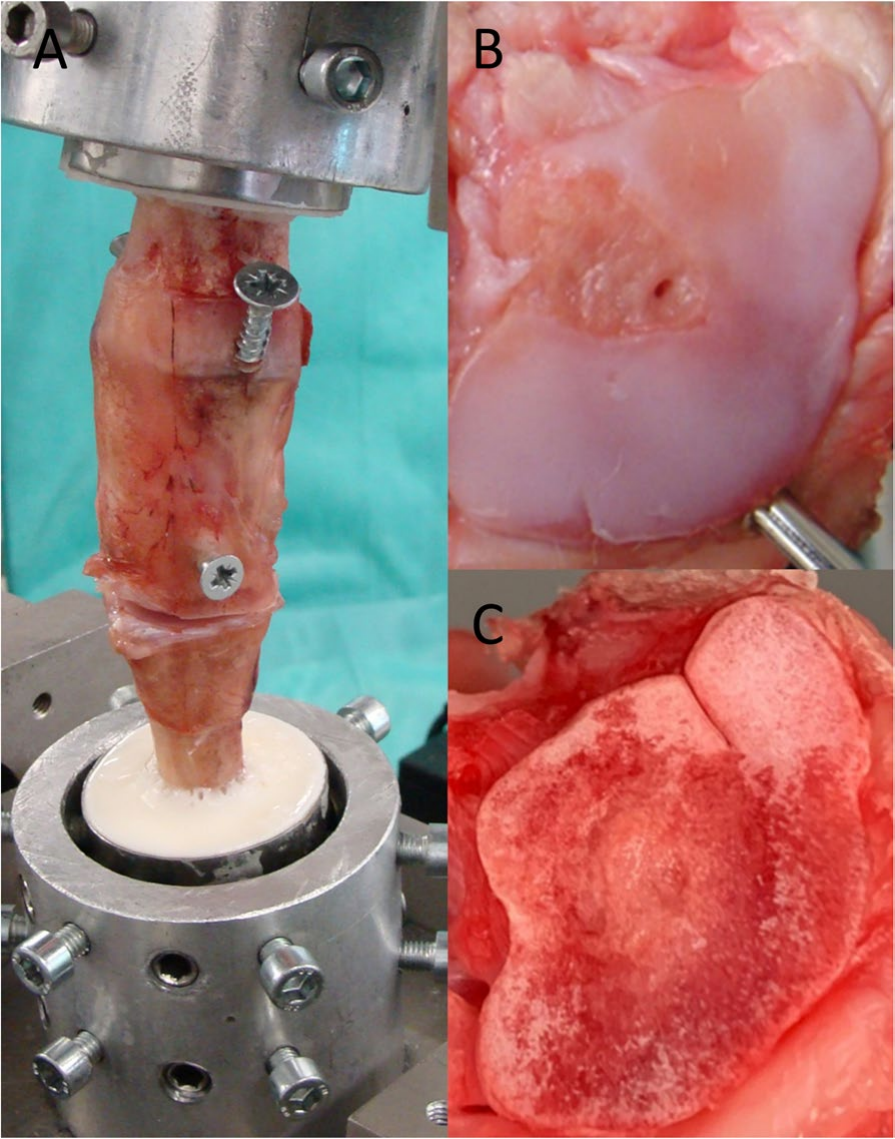
Experimental setup. Embedded and screw-fixed joint before the final application in the test machine (**A**). Native condition of the joint after opening the capsule (**B**). Cartilage defect condition (**C**)

Nine fresh, frozen ovine CMC joints (stored at − 20 ◦C) were used. These joints were obtained directly post mortem and cut 8 cm distal and proximal from the joint center, which was controlled fluoroscopically (Philips Digital Scopofix BV 25, Hamburg, Germany). Prior to testing, the specimens were thawed over 12 h at room temperature and dissected from the skin and musculature. The brachidium joints were fixed with screws to prevent residual movements. Subsequently, the radius shaft and metacarpal bone were embedded in a custom-made metal frame with a two-component resin (Technovit Universal Fluid and Pouder 2060, Heraeus Kulzer GmbH, Wehrheim, Germany). The joint capsule was resected just prior to the start of the measurements to prevent side effects of soft tissue interactions and to avoid influences of the capsule on the DE during the further test process (Fig. 1 A).

### Experimental procedure

**Fig. 2:**
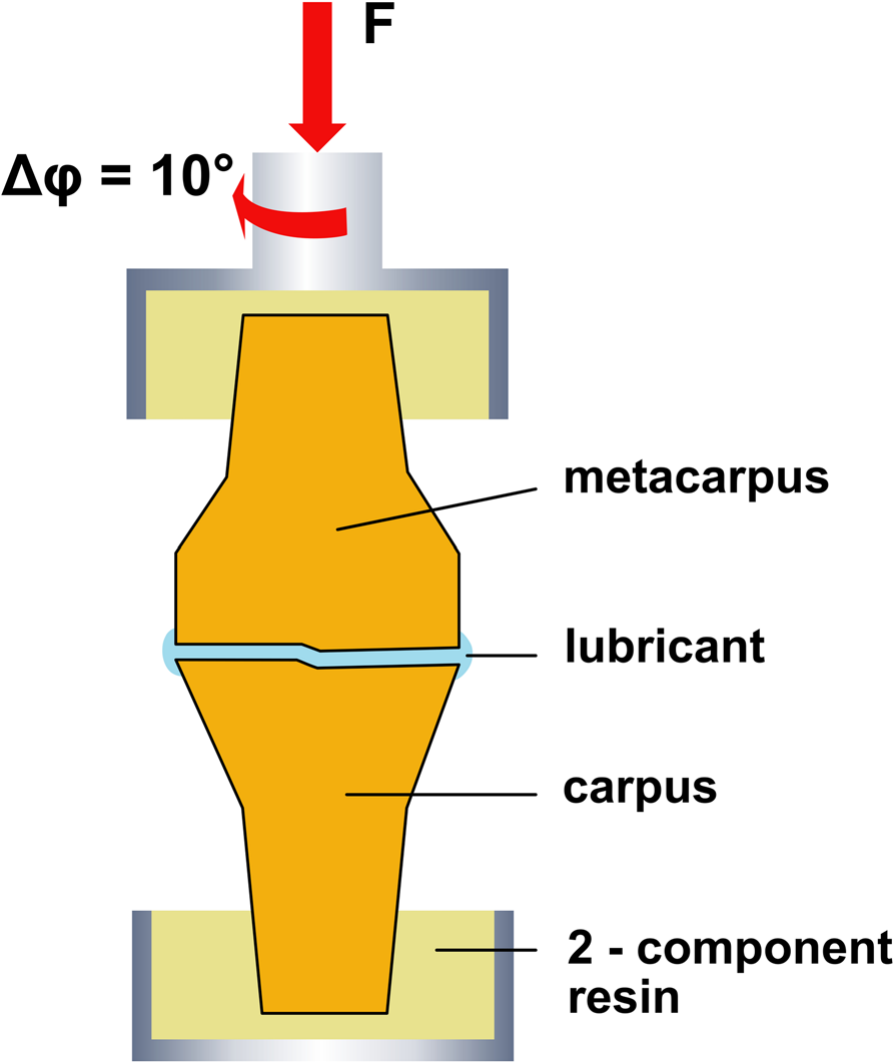
Schematic picture of the experimental setup. The joint is embedded in Polymethylmethacrylat (PMMA) and connected to the material testing machine (MTS) plunge. Joint movement is performed with an angle-controlled triangle shaped rotation (φ_max_ = 10◦) around the long axis (Δφ) under a constant axial preload (F)

**Fig. 3:**
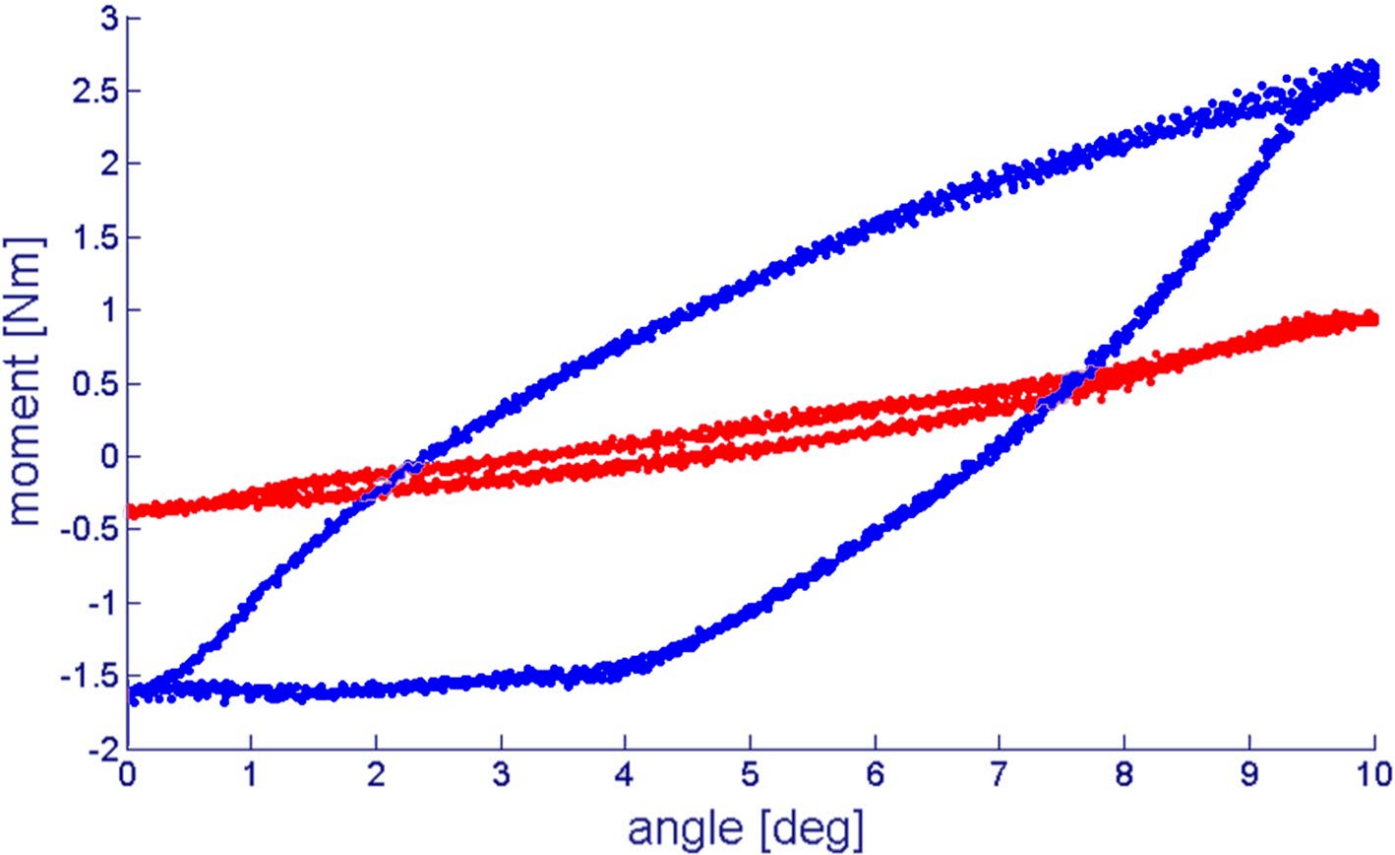
Representative plots for joint motion. Specified angle of rotation (angle [deg]) and measured torque moment (moment [Nm]) in different conditions. The dissipated energy is represented by the area enclosed within the hysteresis curve. Red: *Native* joint and 400 N axial load. Blue: *Cartilage defect* and 400 N axial load

The embedded specimens were mounted to a material testing machine (MTS 858 Mini Bionix, Eden Prairie, MN, USA, 25 kN load cell) directly after opening the capsule (Fig. 1). Because of the open experimental setup, the temperature during the experiment was constantly 22° C. We started the measurements in *native* condition (Fig. 1 B) with a strictly coaxial alignment (see Fig. 2). Corresponding to the physiological joint load in a standing position of the animal, we applied a constant preload of 100 N and an angle-controlled torsional motion up to a 10° magnitude with a triangularly shaped target value and a frequency of 0.5 Hz for 20 cycles (Fig. 2) [23]. As previously shown, 10° rotation angle are the physiological movement of the tested joints [21, 23, 26]. Rotation angle and torque around the longitudinal axis were recorded with a separate hydraulic circuit in the described testing machine to calculate DE, according to a previous study [26] (Fig. 3).

The DE was calculated with formula 1:

1 $$\begin{array}{c}{E}_{dis}= \underset{\varphi }{\overset{}{\oint }}M d\varphi \\ \begin{array}{l}{\text{E}}_{\text{dis}}: \\ M:\\ \varphi :\end{array}\begin{array}{l}\text{dissipated energy}\\ {\text{torque}}\\ {\text{rotation}}\text{ angle}\end{array}\end{array}$$

After finishing 20 cycles, we increased the constant axial force in ascending steps of 100 N up to 400 N at last. Relevant wear caused by the measurements could be excluded in a previous study by long-term control tests [26]. Due to the preconditioning effect, the last 15 cycles were considered for the DE calculation. Five preconditioning cycles are sufficient as shown before, since there is no more significant change in friction properties over time [26]. Measuring a longer time period, especially in defect condition, arising structural damages would distort results.

Simulating cartilage damage, the specimens were dried out for 16 h over night at 8° C. The joint parts were divided and the joint surfaces were neither covered nor moistened. Before carrying out the experiment, the specimens were warmed up again to the experiment temperature of 22 °C. The testing procedure with the parameters mentioned above was performed again in *cartilage damage* condition and data were collected (Fig. 1 C). Then *NaCl* 0.9% (B. Braun Deutschland GmbH & Co. KG, Melsungen, Germany), *Hyaluron* (Ostenil® TRB Chemedica AG, Feldkirchen, Germany, concentration: 10 mg/ml, MW: 1.2–1.4 × 10^6^ Da) or fetal calf serum (*FCS*) (Invitrogen, Karlsruhe, Germany) was applied randomly to the joints by dribbling the lubricant onto the joint, resulting in three joints per substance. After lubrication, the measurement procedure described above was repeated and called *lubricant*.

Since a biotribological test setup is used, a tribological system analysis was performed (according to GfT, Gesellschaft für Tribologie Arbeitsblatt 7/2002 (http://www.gft-ev.de) [27]) (Table 1).

**Table 1:** Tribological system analysis of the experimental setup (according to GfT, Gesellschaft für Tribologie Arbeitsblatt 7/2002 (http://www.gft-ev.de) [27]) (NaCl: Natriumchloride solution; FCS: fetal calf serum; HA: hyaluronic acid)

	**Native condition**	**Cartilage damage condition**	**Relubricated condition**
**Strain collective**			
Form of movement	Sliding	Sliding	Sliding
Sequence of movement	Oscillating	Oscillating	Oscillating
Force	100 – 400 N	100 – 400 N	100 – 400 N
Velocity	5°/s rotation	5°/s rotation	5°/s rotation
Temperature	22 °C	22 °C	22 °C
Exposure time	40 s	40 s	40 s
**Structure of the Tribosystem**			
Body and Counterbody	Cartilage disc (5% cells, 95% matrix containing macromolecules and 80% water)	Cartilage disc (5% cells, 95% matrix containing macromolecules and 80% water)	Cartilage disc (5% cells, 95% matrix containing macromolecules and 80% water)
Intermediate medium	Synovial fluid		NaCl vs. FCS vs. HA
Surrounding medium	Air	Air	Air
**Wear analysis**			
Friction condition	Hydrodynamic and mixed mode friction	Boundary mode friction	Hydrodynamic and mixed mode friction
Type of wear	Abrasion	Abrasion	Abrasion

### Data analysis

DE was calculated using standard integration methods (Simpson method, Matlab R2020a, The Math-Works, Inc., Natick, MA). Group comparison in successive measurements was performed with the repeated measurement ANOVA, similar to previous studies on DE [22]. Normal distribution was assessed by the Shapiro–Wilk test. Further the testing of sphericity was calculated with the Mauchly Test [28]. In case of violations of sphericity the Greenhouse–Geisser adjustment was used for correction [29]. Post-hoc analysis was performed Bonferroni-adjusted. Before analyzing the three different lubricants (*NaCl, FCS* and *Hyaluron*), the values for DE was normalized to the condition *native* with the formula: DE_normalized_ = DE_lubricant_ – DE_native_. For group comparison, a one-way ANOVA with Tukey-HSD as post-hoc test was performed.

## Results

First, we asked whether the dependence of the DE on the axial load, can be reproduced in testing different lubrication conditions. We therefore analyzed and compared the DE for the four different axial load steps (100 N, 200 N, 300 N and 400 N) in the *native* and in the *lubricant* condition. One extreme outliner, presumably triggered by a malfunction of a hydraulic valve, had to be excluded, resulting in *n* = 17 values per group (9 joints in 2 conditions = 18—1 outliner).

**Fig. 4:**
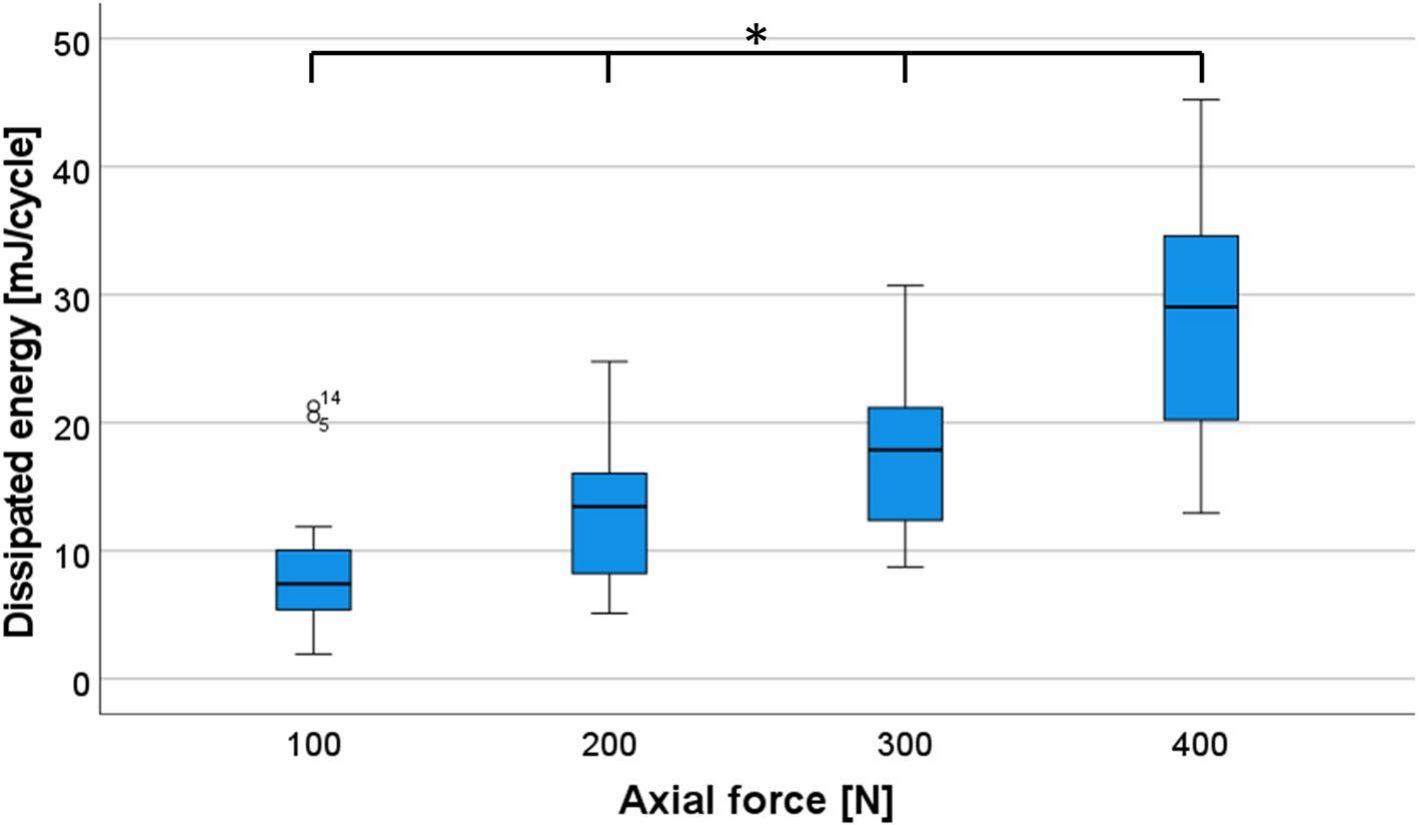
Diagram for dissipated energy (DE) as a function of axial load**.** Boxplots displaying the DE **[**mJ/Cycle**]** measured in the *native* or *lubricant* condition (blue) as a function of axial load. * denotes a significant difference (*p* < 0.001) after repeated measurement ANOVA and post-hoc test with the Bonferroni correction

The DE of the four steps was normally distributed, as assessed by the Shapiro‐Wilk test (*p* > 0.05). To compare the effects of different axial load on DE, data were analyzed with repeated measures ANOVA, detecting significant changes between the four steps (100 N, 200 N, 300 N and 400 N) F(1.13,18.03) = 107.24, *p* < 0.001 (Fig. 4).

In the post-hoc tests with Bonferroni correction, we found significant differences between all axial load steps (*p* < 0.001). In conclusion, we could determine a significant increase in DE with increasing axial load.

**Fig. 5:**
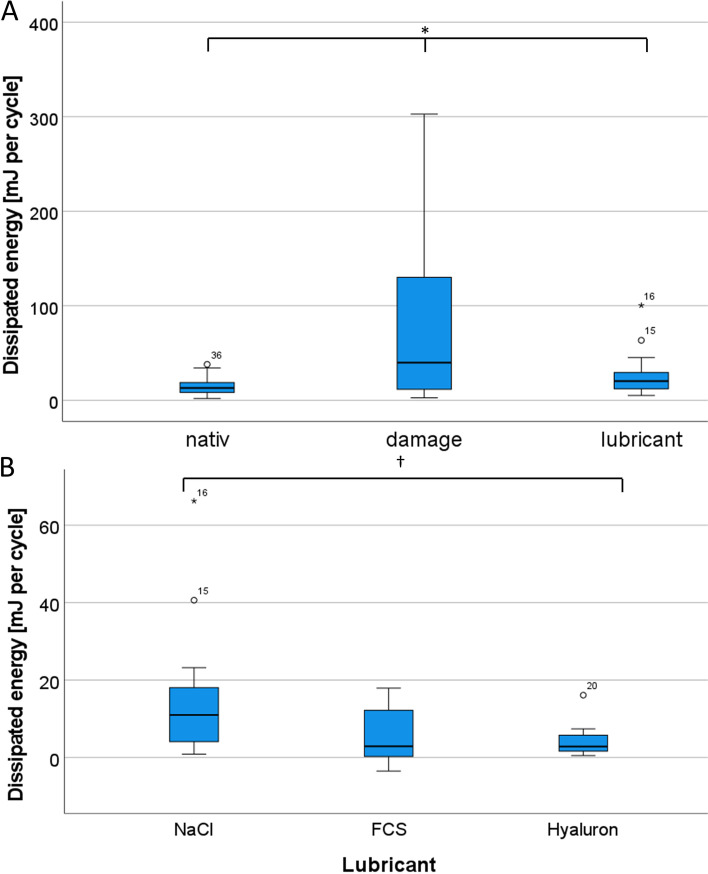
Comparison of dissipated energy (DE). Boxplots (**A**) displaying the DE **[**mJ/Cycle**]** measured in the *native*, *cartilage defect* and *lubricant* condition (* denotes a significant difference (*p* < 0.05) MANOVA with Bonferroni correction) and (**B**) the DE after normalization of the three tested lubricants († denotes a significant difference after one-way ANOVA and post-hoc test)

Second, we investigated whether the DE can be increased through artificial joint damage and then reduced again through lubrication. We therefore compared the values of DE for *native*, *cartilage damage* and *lubricant* under 400 N constant axial preload (Fig. 5 A).

As a result, we could document an increase in DE from *native* (Mean: 15.0 mJ/cycle, SD: 8.98) to *cartilage damage* (M: 74.4 mJ/cycle, SD: 79.02) of 59.4 mJ/cycle (+ 396%). Further, we found a decrease after re-lubrication to 23.6 mJ/cycle (SD: 18.47) of 50.8 mJ/cycle (-68.3

%). Comparing the conditions *native* and *lubrication*, we found only a very small increase of 8.6 mJ/cycle (57.1%) (Table 2).

**Table 2:** Values of dissipated energy (DE) for different conditions

Condition	N	DE Mean [mJ/cyle]	Standard deviation [mJ/cyle]	Minimum [mJ/cyle]	Maximum [mJ/cyle]
*Native*	36	14.99	8.98	1.93	38.03
*Cartilage defect*	36	74.40	79.02	2.65	302.74
*Lubrication*	36	23.57	18.47	5.06	100.42

Again, we performed repeated measures ANOVA with Greenhouse–Geisser adjustment, due to violations of sphericity. Regarding the results, we found significant changes between the three conditions (F(1.03,35,98) = 22.52, *p* < 0.001). In the post-hoc tests, we found significant differences between all conditions (*native* vs. *lubrication: p* = 0.001, all other pairs: *p* < 0.001). In summary, we can confirm both an increase in DE due to the artificial cartilage damage and an almost complete normalization after relubrication.

Finally, we determined whether HA leads to an improved reduction in DE compared to other lubricants. Therefore, we compared the DE values for *NaCl*, *FCS* and *Hyaluron* after normalization (Table 3 and Fig. 5 B). The highest values by far were detected for *NaCl* (M_Norm_ = 16.4 mJ/cycle, SD: 19.14). *Hyaluron* achieved the lowest value (M_Norm_ = 4.3 mJ/cycle, SD: 4.31), although the gap to *FCS* (M_Norm_ = 5.1 mJ/cycle, SD: 7.07) was small (Difference in DE_normalized_: 0.85, 20.0%). For performing a one-way ANOVA, we first checked homogeneity of variances, which was asserted using Levene’s Test. The result demonstrated, that equal variances could be assumed (*p* = 0.117). The one-way ANOVA showed significant changes between the three groups (F (2,33) = 3.79, *p* = 0.033). Regarding the post-hoc tests, significant changes could be found between *NaCl* and *Hyaluron* (*p* = 0.049). In conclusion, the results indicate a superiority of hyaluronic acid as a lubricant compared to NaCl and FCS.

**Table 3:** Differentiated values for lubrication after normalization of dissipated energy (DE) ( DE_normalized_ = DE_lubricant_ – DE_native_)

Lubricant	N	DE Mean [mJ/cyle]	Standard Deviation [mJ/cyle]	Minimum [mJ/cyle]	Maximum [mJ/cyle]
*NaCl*	12	16.38	19.14	0.87	66.28
*Fetal Calf Serum*	12	5.11	7.07	-3.52	17.92
*Hyaluron*	12	4.26	4.31	0.50	16.11

## Discussion

In the present study, we investigated the influence of axial load, cartilage damage and different lubricants on DE in whole ovine CMC joints with physiologic movement in an in-vitro testing model.

Again, we were able to show that increasing axial load leads to increased friction in joints [23]. This is especially true for joints with cartilage damage, where joint friction increased significantly (*p* = 0.001) with increasing axial load. When trying to interpret these results, the knowledge of the influencing factors of the viscosity of HA and its rheological behavior are crucial. Key factors of the viscosity are the molecular weight and concentration of HA as well as shear forces [30]. Therefore, a one possible factor for the higher DE in joints with cartilage damage are the reduced concentration and molecular weight of HA. The increasing DE with higher axial load has to be seen in connection with the non-Newtonian behavior of HA which causes a decreasing viscosity with increasing shear forces [30, 31]. Therefore, higher axial loads are leading to higher shear forces which can cause a decrease in HA-viscosity. Thinking therapeutically, the advice of losing weight to reduce axial load on joints with cartilage damage is confirmed once more.

Furthermore, our results indicate an enormous decrease in friction caused by addition of lubricants, where the values of DE fell to one-third of the initial value. The effect of HA was clearly superior to the other tested lubricants having a 16.7% and 74% lower DE compared to FCS and NaCl, respectively.

The biomechanical consideration of degenerative changes in joints is crucial for the evaluation of therapeutic approaches. Here, the investigation of friction plays an essential role. However, this measurement is not easy to implement, especially since an unphysiological measurement situation arises in the previously known investigation approaches, such as pin-on-disc or pendulum measurements.

However, due to its easy feasibility and high reproducibility, the pin-on-disc measurement is the most widely used investigation tool, resulting in a large number of tribological studies that examined the effect of HA on friction [16–18, 32, 33]. Despite improvements in the system in recent years from the use of synthetic surfaces as counterpart, such as glass or metal, to cartilage on cartilage contacts, the pin and disc method cannot address the physiological situation in the joint [34]. For the measurement, small, flat cartilage discs are removed from the joint and inserted into a tribotester [18]. Three-dimensional surface structures of the cartilage or physiological joint movements cannot be detected. The two major disadvantages can be eliminated by determining the DE as a friction parameter, as these are considered as a result of the experimental setup. Comparing our results in difference between HA and control lubricant with pin on disc studies, a wide range of results could be observed: Bell et al. found a maximum increase of 133.3% in coefficient of friction (COF) comparing HA (COF: 0.12) with control lubricant (phosphate buffered saline (PBS); COF: 0.28) in a static pin-on-disc model with degenerated bovine cartilage [17]. Subsequently, Forsey et al. found an increase of 50% in COF, comparing 10 mg/ml HA (COF: 0.15) with control lubricant (Ringers solution, COF: 0.3) in human degenerated cartilage samples from patients undergoing total joint replacement surgery in a cartilage-on-cartilage sliding model [18]. In our study, DE_normalized_, was a quarter in HA (4.26) compared with control lubricant (*NaCl*: 16.38).

A further published testing approach is the pendulum, offering the possibility to investigate whole joints similar to the DE. However, despite the applied pendulum oscillation may simulate joint movement, the forces occurring in vivo differ significantly. An example for the investigation of HA using a pendulum model is a study published by Kawai et al. [19]: They tested different types of HA with increasing MW from 1.0 to 2.3 × 10^6^ Da and analyzed the COF of temporomandibular joints in pigs. Comparable to our investigations, cartilage damage was simulated by a simple model, in this study by scouring with gauze. Comparing the results, Kawai et al. found an increase in COF from *native* (COF: 0.0164) to *cartilage damage* (COF: 0.0398) of 143%, which was in our study 396% (DE_native_: 14.99 to DE_cartilage damage_: 74.40). Regarding HA with similar MW (Kawai et al.: 1.7 × 10^6^ Da vs. our data: 1.2 to 1.6 × 10^6^ Da), in both studies an increased friction value after relubrication was found compared to *native* (Kawei et al.: 145% of *native*, our data: 157% of *native*) [19]. The fact, that the friction is increased although the system is relubricated has to be seen from a tribological but also a biochemical point of view. In our experimental setup the relubricated joints were prior tested with cartilage damage simulated by drying out. In this setup, a high friction with enhanced abrasion has to be assumed causing surface damage. When the specimens are relubricated, the surface is still critically impaired leading to a higher friction. Furthermore, the impact of influence of other lubricants on friction such as PRG4 still present in native joints cannot be determined exactly. It must therefore be assumed that the addition of HA leads to interactions with other lubricants and thus HA does not exclusively lead to reduced friction. However, the composition of the lubricant after drying out has changed and therefore it has to be assumed, that the interaction of the components of the lubricant has changed as well. This can also be an explanation why FCS, although containing macromolecules, too, does not limit the friction to the same extend as HA.

Another approach was described by Obara et al., applying a translational movement to the knee joint and measuring the friction by a robot system [20]. Strengths of Obara's study is the use of an in vivo model (rabbits) and induction of the osteoarthritis with ACL resection and papain injection. However, the physiological sequence of movements with a complex rolling sliding movement cannot be considered. Compared to our study with a decrease in friction of 68% after application of HA, Obara was only able to record a discrete decrease of approx. 10% [20].

What has to be kept in mind for all kinds of lubrication experiments is the lubrication mode visualized by Stribeck curve. The lubrication mode is dependent on strain and speed of movement [35]. According to preliminary investigations, at least a mixed mode or even hydrodynamic friction can be assumed in our experimental setup. A stick–slip mechanism is only conceivable at the turning points [35, 36].

When talking about the lubrication capability of solutions, the intrinsic viscosity and its influencing factors like pH range, osmotic pressure or temperature are crucial [37]. Since we used ready-made products, we were not able to influence pH range or osmotic pressure. However, experimental temperature was variable. It could be shown that for macromolecular solutions, especially HA, that through a changed conformation and steric of the molecular chains the viscosity decreases with increasing temperature. If the temperature changes from 25 to 65° C, the viscosity decreases by around 25% [30, 38]. With the constant temperature of 22 °C we have chosen, we guaranteed the same conditions for all lubricants and an almost realistic temperature of the lower extremity [39].

An important prerequisite for the biomechanical testing of lubricants in joints is the induction of osteoarthritis. Several induction methods have been developed in recent years. Inducing a nearly realistic cartilage damage in living animals by severing the anterior cruciate ligament (ACL) seems to be the most adequate procedure. Talking about limitations, the induction of cartilage damage by drying out over night is not as realistic as severing the ACL or by an injection of papain [20, 40–42]. However, these procedures are very tedious and could not be realized in the in vitro situation, especially since the test specimen disintegrates beforehand [43]. Therefore, based on previous pin-on-disc studies on animal cartilage, we simulated cartilage damage using a drying process for biomechanical testing [44, 45].

One clear strength of the present study is the experimental workup. Having complete joints with physiological movements, a more reliable statement regarding joint friction is possible. Simplifying effects of pin-on-disc or pendulum models can be ruled out as already described.

## Conclusion

To the best of our knowledge, with this experimental setup we present the first characterization of different lubricants on complete joints with physiological movements. Taken together, we were able to elucidate three effects in joints with cartilage damage. First, the friction in damaged joints increases significantly compared to native joints. Second, especially in damaged joints, the friction increases significantly more with increased axial load compared to native or lubricated joints. Our most important finding, which is in line with existing literature, is that lubricants can achieve an enormous decrease in friction. Comparing different lubricants, our results indicate the highest decrease in friction for HA.

However, it is important to keep in mind that not only HA but also other lubricants are crucial for the reduction of friction in synovial joints.

## Data Availability

The datasets used and/or analyzed during the current study are available from the corresponding author on reasonable request.

## Abbreviations

ACL: Anterior cruciate ligament
CMC: Carpo-metacarpal
COF: Coefficient of friction
DE: Dissipated energy
FCS: Fetal calf serum
HA: Hyaluronic acid
MW: Molecular weight
NaCl: Natrium chlorid
OA: Osteoarthritis
PBS: Phosphate buffered saline
PMMA: Polymethylmethacrylat
PRG4: Proteoglycan 4
SAPL: Surface-active phospholipids
SF: Synovial fluid
